# The nucleolar-related protein Dyskerin pseudouridine synthase 1 (DKC1) predicts poor prognosis in breast cancer

**DOI:** 10.1038/s41416-020-01045-7

**Published:** 2020-09-01

**Authors:** Khloud A. Elsharawy, Omar J. Mohammed, Mohammed A. Aleskandarany, Ayman Hyder, Hekmat L. El-Gammal, Mohamed I. Abou-Dobara, Andrew R. Green, Leslie W. Dalton, Emad A. Rakha

**Affiliations:** 1 Nottingham Breast Cancer Research Centre, Division of Cancer and Stem Cells, School of Medicine, University of Nottingham Biodiscovery Institute, University Park, Nottingham, UK; 2 Faculty of Science, Damietta University, Damietta, Egypt; 3 Department of Histopathology, South Austin Hospital, Austin, TX USA

**Keywords:** Breast cancer, Oncology

## Abstract

**Background:**

Hypertrophy of the nucleolus is a distinctive cytological feature of malignant cells and corresponds to aggressive behaviour. This study aimed to identify the key gene associated with nucleolar prominence (NP) in breast cancer (BC) and determine its prognostic significance.

**Methods:**

From The Cancer Genome Atlas (TCGA) cohort, digital whole slide images identified cancers having NP served as label and an information theory algorithm was applied to find which mRNA gene best explained NP. Dyskerin Pseudouridine Synthase 1 (*DKC1*) was identified. *DKC1* expression was assessed using mRNA data of Molecular Taxonomy of Breast Cancer International Consortium (METABRIC, *n* = 1980) and TCGA (*n* = 855). DKC1 protein expression was assessed using immunohistochemistry in Nottingham BC cohort (*n* = 943).

**Results:**

Nuclear and nucleolar expressions of DKC1 protein were significantly associated with higher tumour grade (*p* < 0.0001), high nucleolar score (*p* < 0.001) and poor Nottingham Prognostic Index (*p* < 0.0001). High DKC1 expression was associated with shorter BC-specific survival (BCSS). In multivariate analysis, *DKC1* mRNA and protein expressions were independent risk factors for BCSS (*p* < 0.01).

**Conclusion:**

DKC1 expression is strongly correlated with NP and its overexpression in BC is associated with unfavourable clinicopathological characteristics and poor outcome. This has been a detailed example in the correlation of phenotype with genotype.

## Background

Breast cancer (BC) is the most common cancer diagnosed in women worldwide, accounting for ~1 in 10 new cancer diagnoses each year and is the second most common cause of death^1,2^ due to cancer. BC is a heterogeneous disease with variable morphologies and response to therapy. Some morphological features, especially histological grade, have been well validated to have a strong prognostic value and their assessment helps in prognostic stratification of BC patients for treatment decisions.^3^

In the Nottingham cohort, nucleolar prominence (NP) has recently been shown to be a significant predictor for patient outcome as well as of being highly correlated with tumour grade. Since the NP is a distinctive morphological attribute, it is hypothesised to possibly serve as a substitute for the highly subjective pleomorphism component score of the Nottingham BC grading.^4^ Consequently, it is deemed imperative to explore the correlations between the nucleolar phenotype and genotype.

The major function of the nucleolus is synthesis and assembly of ribosomes^5^ where both are associated with malignant transformation and cancer progression.^6,7^ Indeed, ribosome biogenesis depends on the cancer growth rate, which is directly related to nucleolar size of malignant cells. Nucleolar size and cell kinetics’ parameters are interrelated because of the increasing rate of ribosome biogenesis in proliferating cells.^8^ In some solid cancer and haematological malignancies, the ribosome biogenesis rate increases as a consequence of overexpression of the oncogene c-Myc, which controls all the steps of ribosome biogenesis.^9^ Despite the biological and clinical significance of NP in BC, the key gene associated with NP and its prognostic significance remains to be defined.

Dyskerin Pseudouridine Synthase 1 (DKC1) is a predominantly nucleolar protein encoded by *DKC1* gene and mapped at Xq28.^10^ DKC1 is a crucial component of the telomerase complex and is required for normal telomere maintenance and post-transcriptional processing of precursor rRNA. Therefore, DKC1 is necessary for tumour cell progression through mechanisms related to its function in the processing of rRNA precursor.^11^ Usually, clinically indolent and slow-growing tumours express lower levels of DKC1 and its inhibition slows or hinders the proliferation in most cell types.^11,12^ Through various deprivation of function approaches, emerging evidence suggests that DCK1 may regulate other cellular processes, including IRES-mediated translation, telomere maintenance independent of telomere length regulation, mitosis, transcription and possibly microRNA processing.^13,14^ Upregulation of DKC1 expression has been reported in several human cancers including hepatocellular carcinoma,^15^ neuroblastoma,^16^ lymphoma,^17^ melanoma,^18^ prostate cancer,^19^ colorectal cancer^20^ and ovarian carcinoma.^21^

## Methods

### Principle of DKC1 selection

We have applied an information theory (IT) approach to The Cancer Genome Atlas (TCGA) breast cancer dataset. The IT approach was used for feature selection to identify the key gene associated with NP, which was assessed morphologically in full face invasive BC sections stained with haematoxylin and eosin (H&E) using digital whole slide images (WSI) as explained in our previous study.^4^ The TCGA BC cohort was employed since it contains satisfactory whole slide images and mRNA-seq2 data present in 743 cancers. In the IT approach utilised, the nucleolar score served as a label and 20,339 mRNA transcripts served as predictor variables. The IT algorithm is a ‘greedy’ algorithm and reduces the number of features selected. “Greedy” is the term used in the machine learning community to describe an algorithm, which selects the optimal feature at each step and does not alter any choices already made based on findings from future choices.^22^ The analysis showed that the attribute, which exhibited the highest mutual information (information gain) with NP, was *DKC1*. Moreover, the detection of *DKC1* was supported by LASSO regression feature selection. The required functions for LASSO were obtained from R library Glmnet.^23^ LASSO regression is also capable of reducing the number of predictors and thereby allowing for a focused study of a few attributes.^24^ Therefore, by applying the IT approach and LASSO regression, the selection was limited to a single gene (*DKC1*). This was followed by evaluating *DKC1* mRNA and DKC1 protein expression in large clinically annotated cohorts of BC to evaluate its clinicopathological and prognostic value in invasive BC as described below.

### Study cohorts for transcriptomic analysis

The discovery of *DKC1* was by study of TCGA cohort. The TCGA was also used to assess the possible correlation between *DKC1* mRNA expression and the variables recorded in this cohort.^25^ The Molecular Taxonomy of Breast Cancer International Consortium (METABRIC) cohort (*n* = 1980) was used to evaluate *DKC1* gene copy number (CN) aberrations and gene expression.^26^ Genomic and transcriptomic data for the METABRIC cohort had been obtained using the Affymetrix SNP 6.0 and Illumina HT-12v3 platforms, respectively.^26^ The association between *DKC1* mRNA expression, copy number aberrations and clinicopathological parameters, molecular subtypes and patient outcome was investigated. Breast Cancer Gene Expression Miner online dataset v4.3 (http://bcgenex.centregauducheau.fr/BC-GEM/GEM-requete.php) was also used as external validation of *DKC1* mRNA expression.

### Study cohort for protein expression

The Nottingham BC patient cohort was used to evaluate the immunohistochemical (IHC) expression of DKC1. This cohort is a well-characterised large series (*n* = 943) of invasive BC patients aged ≤70 years and presented at Nottingham City Hospital between 1999 and 2006. The cohort has long-term clinical follow-up and clinicopathological data included patient’s age at diagnosis, histological tumour type, tumour grade, tumour size, lymph node status, Nottingham Prognostic Index (NPI) and lymphovascular invasion (LVI). Patient outcome data were obtained including BC-specific survival (BCSS), defined as the time (in months) from the date of primary surgical treatment to the time of death from BC and distant metastasis free survival (DMFS) defined as time (in months) from primary surgical treatment until the first event of distant metastasis. Patients were treated based on tumour features, NPI and hormone receptor status. Endocrine therapy was given to patients who had oestrogen receptor positivity (ER+) tumours with high NPI scores (>3.4), whereas no adjuvant therapy was given to patients with ‘good’ NPI scores (≤3.4). Premenopausal patients with moderate and poor NPI scores were candidates for chemotherapy, while postmenopausal patients with ‘moderate’ or ‘poor’ NPI scores were given hormonal therapy only. Classical treatment of cyclophosphamide, methotrexate and fluorouracil (CMF) was used as a therapy for patients presented with absence of ER expression and clinically fit to receive chemotherapy. None of the patients in the current study cohort received neoadjuvant therapy. Data related to the expression of ER, progesterone receptor (PR) and human epidermal growth factor receptor 2 (HER2) as well as Ki67 were available.^27–30^ Molecular subtypes were based on tumour immunohistochemical (IHC) profile and the Elston–Ellis^31^ mitotic score as ER+/HER2−; low proliferation (mitotic score 1), ER+/HER2− high proliferation (mitotic scores 2 and 3), HER2-positive class: HER2+ regardless of ER status, TN: ER−, PR− and HER2−.^32^ There was no significant differences in the distribution of the clinicopathological parameters between the Nottingham and the METABRIC cohorts (all correlation coefficients ≥0.948, all *p* < 0.0001)^33^ (Supplementary Table 1).

### DKC1 validation by western blotting

The antibody specificity of anti-DKC1 antibody (EPR10399, Abcam, UK) was validated using western blotting (WB) performed on cell lysates of a wild and transfected MDA-MB-231 human breast cancer cell line (American Type Culture Collection; Rockville, MD, USA). The forward transfection of siRNA procedure was followed according to *DKC1* siRNAs manufacturer’s instructions. In brief, the cells were seeded in 6-well plate at a cell density of 3 × 10^5^ cells per well and incubated overnight in 37 °C 5% CO_2_ incubator. The following day, the cells reached about 40% confluence and were transfected with 10 nM of three different IDs of *DKC1* siRNA (Cat#:4392420, ThermoFisher Scientific, UK). A transfection with 10 nM scrambled siRNA sequence (Cat#:4390843, ThermoFisher Scientific, UK) was carried out in the experiment and considered as a negative control. DKC1 protein expression of untransfected & transfected cells was then determined by the Western blotting. Briefly, after collecting the cell lysates, a dilution of 1:1000 of the primary antibody and 1:15000 IRDye 800CW Donkey anti-rabbit secondary antibody (LI-COR Biosciences) were applied, and 5% milk /PBS-Tween (0.1%) (Marvel Original Dried Skimmed Milk, Premier Food Groups Ltd., UK) was used for blocking and antibodies incubation. Mouse monoclonal anti-β-actin primary antibody (1:5000) (Sigma–Aldrich, UK) with IRDye 800CW Donkey anti-mouse fluorescent secondary antibody (LI-COR Biosciences) were used to visualise a marker of endogenous control. Visualisation of *DKC1* band was done by using the Odyssey Fc with Image Studio 4.0 (LI-COR Biosciences).

### Tissue microarrays and immunohistochemical analysis

Invasive BC tissues were previously arrayed as tissue microarrays (TMA) using the Grand Master^®^ (3D HISTECH^®^, Budapest, Hungary).^34^ IHC staining was performed on 4 μm TMA thick sections using the Novocastra Novolink™ Polymer Detection Systems kit (Code: RE7280-K, Leica, Biosystems, Newcastle, UK). Antigen retrieval was performed in citrate buffer pH 6.0 using a microwave (Whirlpool JT359 Jet Chef 1000 W) for 20 min. Rabbit monoclonal DKC1 was diluted at 1:50 in Leica antibody diluent (RE AR9352, Leica, Biosystems, UK) and incubated with the sections for 60 min at room temperature. A negative control was obtained by omitting the incubation with primary antibody while formalin fixed placenta tissue was used as a positive control according to manufacturer’s datasheet.

### Assessment of DKC1 protein expression

Scanning of TMA stained sections into high-resolution digital images was performed by using a NanoZoomer scanner (NanoZoomer; Hamamatsu Photonics, Welwyn Garden City, UK) at ×20 magnification. Scoring of DKC1 nuclear and nucleolar^4^ expression was evaluated based on a semi-quantitative scoring using modified histochemical score (H-score), where the intensity of staining was multiplied by the percentage of positive cells in the tissue for each intensity, producing a score ranging between 0 and 300.^35^ A score index of 0, 1, 2 and 3 corresponding to negative, weak, moderate and strong respectively were used for intensity. The percentage (0–100) of positive cells for each intensity was evaluated subjectively. All non-representative cores including folded tissue during processing and staining, cores with only normal breast tissue and cores with invasive tumour <15% of core surface area were excluded from scoring. All the cores were scored by a trained observer (K. Elsharawy) blinded of histopathological and patient outcome data. Further, to test the interobserver’s reproducibility of the scoring, a subset of TMA cores (10%) was randomly selected and double scored by a second trained observer (M. Aleskandarany). Moreover, for further evaluation of scoring reproducibility, 20% of the cases were double scored by the main observer (K. Elsharawy) after 5 months washout period blind from the first scores.

### Statistical analysis

IBM-SPSS statistical software 24.0 (SPSS, Chicago, IL, USA) was used in statistical analysis. Interobserver agreement in DKC1 IHC scoring was assessed using intraclass correlation coefficient (ICC). Dichotomisation of DKC1 proteomic and transcriptomic levels expression was determined based on the prediction of BCSS using X-tile bioinformatics software version 3.6.1 (School of Medicine, Yale University, New Haven, USA).^36^ The H-scores of 110 and 10 were the optimal cut-off values of DKC1 nuclear and nucleolar protein expression. Continuous data of *DKC1* mRNA and DKC1 protein expression were used to assess the association with clinicopathological parameters. Differences between three or more groups were investigated using one-way analysis of variance (ANOVA) with the post-hoc Tukey multiple comparison test (for parametric data) or Kruskal–Wallis test (for non-parametric distribution). Student *t-*test (parametric data) or Mann–Whitney test (non-parametric distribution) were used to evaluate the differences between two groups. Spearman’s correlation coefficient was calculated to examine the association between continuous variables. Univariate analysis was visualised using Kaplan–Meier curves and significance was assessed by log-rank test. Cox’s proportional hazard regression models were built for the multivariate survival analysis to adjust for confounding factors. *P* values were adjusted by using Bonferroni correction for multiple testing. For all tests, *p* value < 0.05 was considered as statistically significant. This study followed the reporting recommendations for tumour markers prognostic studies (REMARK) criteria.^37^

## Results

In this study, we have applied the bespoke bioinformatics tools to identify the key genes associated with NP and this identified *DKC1* as the target gene. Then, DKC1 was investigated at the transcriptomic, genetic and protein levels.

### *DKC1* mRNA expression and CN aberrations

**Fig. 1:**
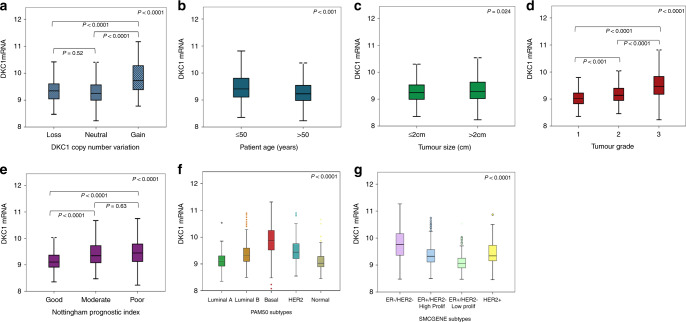
*DKC1* mRNA expression and its association with copy number variations, clinicopathological parameters and molecular subtypes **a** *DKC1* and gene copy number variations **b** *DKC1* and patient age. **c** *DKC1* and tumour size. **d** *DKC1* and tumour grade **e** *DKC1* and Nottingham prognostic index. **f** *DKC1* and PAM50 BC subtypes **g** *DKC1* and SMCGENE subtypes in the METABRIC cohort using one-way analysis of variance with the post-hoc Tukey test.

High DKC1 mRNA expression (log2 intensity >9.4) was observed in 709/1970 (36%) of the METABRIC cases. In all, 77/1980 (4%) of cases showed *DKC1* CN gain, whereas 115/1980 (6%) showed a CN loss. A significant association was observed between *DKC1* CN variation and DKC1 mRNA expression (*p* < 0.0001) **(**Fig. 1a).

### DKC1 protein expression in breast cancer

**Fig. 2:**
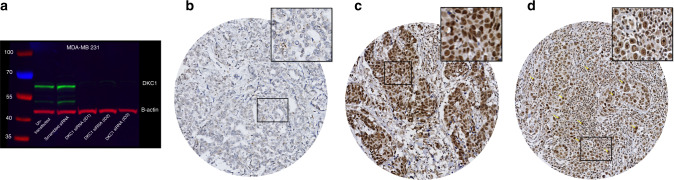
This figure shows DKC1 immunohistochemistry in breast cancer. **a** A representative western blotting for DKC1 expression in cell lysate of MDA-MB-231 breast cancer cell line with the lanes (from left to right) of untransfected, transfected with scrambled siRNA and transfected with three different IDs ([ID1 represents ID s4110], [ID2 represents ID s4111] and [ID3 represents ID s4112]) of *DKC1* siRNA **b** Negative DKC1 IHC expression **c** Positive DKC1 IHC nuclear expression in invasive breast cancer TMA cores and **d** Positive DKC1 IHC nucleolar expression.

Prior to IHC staining, the specificity of the antibody used was validated using WB performed on a *DKC1* siRNA transfected BC cell line. A specific band at the predicted DKC1 molecular weight (58 kDa) was detected for proteins extracted from untransfected cells and those transfected with scrambled siRNA sequences. In addition, the DKC1 band intensity was significantly reduced with proteins extracted from *DKC1* siRNAs transfected cells, confirming the specificity of the antibody utilised. A single band was observed at β-actin molecular weight (42 kDa) demonstrating the uniformity of loaded protein quantities (Fig. 2a).

DKC1 protein expression was observed in the nucleus and nucleoli of invasive BC cells, with expression levels varying from absent to strong (Fig. 2b–d). Strong concordance was observed between the two observers in DKC1 immuno-scoring in 10% of the cases (ICC = 0.864, *p* < 0.0001 for nuclear expression and ICC = 0.781, *p* < 0.001 for nucleolar expression). Moreover, second scoring of 20% of cases after 5 months washout period confirmed concordance ((ICC = 0.822, *p* < 0.0001 for nuclear expression and ICC = 0.804, *p* < 0.0001 for nucleolar expression). At the optimal DKC1 cut-off values (H-score 110 and 10, respectively), High DKC1 nuclear and nucleolar expression were observed in 574/942 (61%) and 153/942 (16%) of the informative tumours, respectively. There was a significant positive correlation between DKC1 nuclear and nucleolar expression (*n* = 429) (correlation coefficient = 0.143, *p* < 0.0001).

### Correlation of *DKC1* mRNA and protein expression with clinicopathological parameters

High *DKC1* mRNA expression was significantly associated with younger patient age, larger tumour size, higher tumour grade and poorer NPI (*p* < 0.001, *p* = 0.024, *p* < 0.0001 and *p* < 0.0001) as shown in Fig. 1b-e, respectively. These associations were confirmed using the Breast Cancer Gene-Expression Miner v4.3 (Supplementary Fig. 1A-C)

In the TCGA BC dataset, similar associations, as described above, were observed with clinicopathological parameters. In particular, high DKC1 mRNA expression was significantly associated with high nucleolar score 3^4^ (*p* < 0.0001, Supplementary Table 2).

High expression of DKC1 protein whether in the nucleus and/or nucleoli was associated with aggressive features of BC including higher tumour grade (*p* < 0.0001), larger tumour size (*p* = 0.04 only with nucleolar expression), higher mitotic scores (*p* < 0.0001), increased nuclear pleomorphism (*p* < 0.0001), higher scores of nucleolar prominence (*p* < 0.001), poor NPI (*p* < 0.0001) and the invasive ductal no special histological type (NST) (*p* < 0.0001), Table 1.

**Table 1:** Clinicopathological associations of DKC1 protein nuclear and nucleolar expression in Nottingham cohort.

Parameters	DKC1 nuclear protein expression			Adjusted *p* value	DKC1 nucleolar protein expression			Adjusted *p* value
	Number (%)	Mean rank	*p* value		Number (%)	Mean rank	*p* value	
Patient age								
≤50	348 (38)	497.8	0.003	**0.027**	348 (38)	477.3	0.09	0.27
>50	580 (62)	444.5			580 (62)	456.8		
Tumour size								
≤2 cm	575 (62)	446.7	0.009	0.7	575 (62)	452.7	0.01	**0.04**
>2 cm	353 (38)	493.4			353 (38)	483.7		
Lymphovascular invasion								
Negative	635 (68)	447.9	0.005	**0.012**	635 (68)	463.1	0.71	0.71
Positive	293 (32)	500.4			293 (32)	467.7		
Axillary nodal stage								
Stage 1	572 (62)	461.6	0.35	0.35	572 (62)	464.7	0.1	0.2
Stage 2	261 (28)	457.4			261 (28)	451.7		
Stage 3	95 (10)	501.1			95 (10)	497.8		
Nottingham prognostic index								
Good	281 (31)	379.3	3.5 × 10^−9^	**<0.0001**	281 (31)	416.8	0.000004	**<0.0001**
Moderate	489 (53)	492.1			489 (53)	476.5		
Poor	150 (16)	509.4			150 (16)	490.2		
IHC subtypes								
ER+/HER2− low proliferation	390 (42)	389.4	1.5 × 10^−10^	**<0.0001**	390 (42)	406.3	9.2 × 10^−21^	**<0.0001**
ER+/HER2− high proliferation	272 (30)	521.1			272 (30)	453.1		
HER2+	115 (13)	504.8			115 (13)	531.7		
Triple negative	140 (15)	494.8			140 (15)	557.6		
Histological subtypes								
Lobular	63 (7)	383.9	4.9 × 10^−7^	**<0.0001**	63 (7)	393.7	7.5 × 10^−7^	**<0.0001**
Tubular	128 (14)	357.1			128 (14)	409.1		
No special type (NST)	623 (67)	497.4			623 (67)	486.3		
Mixed NST and lobular	55 (6)	440.9			55 (6)	416.9		
Other special type	59 (6)	458.4			59 (6)	474.1		
Tumour grade								
Grade 1	105 (11)	269.1	9.8 × 10^−20^	**<0.0001**	105 (11)	402.6	7.8 × 10^−17^	**<0.0001**
Grade 2	392 (42)	442.9			392 (42)	421.8		
Grade 3	432 (47)	532.6			432 (47)	519.4		
Nuclear pleomorphism								
Score 1	10 (1)	224.5	6.9 × 10^−9^	**<0.0001**	10 (1)	375.5	2.8 × 10^−10^	**<0.0001**
Score 2	239 (26)	382.8			239 (26)	396.3		
Score 3	669 (73)	490.4			669 (73)	483.3		
Mitosis								
Score 1	421 (46)	385.2	4.9 × 10^−16^	**<0.0001**	421 (46)	411.1	6.6 × 10^−15^	**<0.0001**
Score 2	188 (20)	476.1			188 (21)	468.5		
Score 3	308 (34)	549.3			308 (33)	518.7		
Tubule formation								
Score 1	46 (5)	296.7	0.000007	**0.0006**	46 (5)	395.8	0.000012	**0.0001**
Score 2	259 (28)	436.9			259 (28)	425.1		
Score 3	613 (67)	481.2			613 (67)	478.9		
Nucleoli								
Score 1	300 (39)	346.2	0.0001	**0.025**	300 (39)	333.7	7.8 × 10^−33^	**<0.0001**
Score 2	334 (43)	411.6			334 (43)	383.5		
Score 3	143 (18)	425.7			143 (18)	517.9		

*P* values in bold means statistically significant.

### DKC1 expression and other markers

The correlation of *DKC1* mRNA with other relevant genes was investigated using the METABRIC and TCGA datasets. The genes were chosen based on published information, being either regulatory genes or those that share or support *DKC1* biological function especially those primarily involved in the ribosomal biogenesis. *DKC1* was positively associated with *GAR1* (*p* < 0.0001), *NOP10* (*p* < 0.001) and *NHP2* (*p* < 0.0001). Moreover, there was a significant association between *DKC1 MKI67* and the regulatory genes *c-Myc* (all *p* < 0.001) (Supplementary Table 3). High *DKC1* mRNA expression was associated with those tumours, which showed *TP53* mutations (*p* < 0.0001, Supplementary Table 4). Also, the statistical analysis showed a significant positive association of high DKC1 protein expression with high Ki67 (χ^2^ = 8.815, *p* = 0.003).

### *DKC1* mRNA and protein expression in BC molecular subtypes

At the transcriptomic level in METABRIC cohort, high *DKC1* expression was significantly associated with hormone receptor negative (ER− and PR−), HER2+ tumours and TNBC (all *p* < 0.0001) as shown in Supplementary Table 4. Similar results were observed upon analysing the publicly available gene-expression data available on the Breast Cancer Gene-Expression Miner v4.3 online platform (Supplementary Fig. 1D–G) and TCGA datasets (Supplementary Table 4).

Regarding the association with the intrinsic PAM50 subtypes,^38^ high expression of *DKC1* mRNA was observed in basal-like, HER2+ and Luminal B tumours (Fig. 1f, *p* < 0.0001). These findings were confirmed using the Breast Cancer Gene-Expression Miner v4.3 (Supplementary Fig. 1H). In the SCMGENE subtypes, high expression of *DKC1* mRNA was observed in the ER−/HER2− cases followed by ER+/HER2− high proliferation class (*p* < 0.0001, Fig. 1g).

DKC1 nuclear and nucleolar protein expression was associated with negative ER status (*p* = 0.04 and *p* < 0.0001 respectively). Moreover, DKC1 nucleolar protein showed a significant correlation within HER2+ and triple negative (TN) tumours (both *p* < 0.0001), Table 2.

**Table 2:** Association of DKC1 protein expression and the expression of other molecular biomarkers in the Nottingham cohort.

Parameters	DKC1 nuclear protein expression			Adjusted *p* value	DKC1 nucleolar protein expression			Adjusted *p* value
	Number (%)	Mean rank	*p* value		Number (%)	Mean rank	*p* value	
Oestrogen receptor								
Negative	182 (20)	511.1	0.01	**0.04**	182 (20)	569.9	3.3 × 10^−18^	**<0.0001**
Positive	748 (80)	454.4			748 (80)	440.1		
Progesterone receptor								
Negative	362 (39)	462.9	0.92	0.92	362 (39)	509.7	4.7 × 10^−10^	**<0.0001**
Positive	565 (61)	464.6			565 (61)	434.7		
HER2 status								
Negative	815 (88)	458.5	0.034	0.1	815 (88)	455.1	0.000003	**<0.0001**
Positive	115 (12)	514.7			115 (12)	539.4		
Triple negative status								
Non-triple negative	789 (85)	458.5	0.058	0.12	789 (85)	446.7	5.4 × 10^−14^	**<0.0001**
Triple negative	141 (15)	504.6			141 (15)	570.9		

*P* values in bold means statistically significant.

There was a higher protein expression of DKC1 (nuclear & nucleolar) in the ER + high proliferative tumours than in the other molecular subtypes (*p* < 0.0001) as shown in Table 1.

### Correlation of DKC1 mRNA and protein expression with patient outcome

In METABRIC cohort, high *DKC1* mRNA expression was associated with poor BCSS in all cases (HR = 1.5, 95% CI = 1.3–1.8; *p* < 0.0001). Moreover, *DKC1* mRNA expression was predictive of BCSS only in luminal B cases (HR = 1.5, 95%CI = 1.1–2.1; *p* = 0.015) as shown in Supplementary Fig. 2A–E. The relationship between high *DKC1* mRNA expression and poor patient outcome in ER+ disease, but not ER− disease, was shown using the TCGA cohort (Supplementary Fig. 3).

**Fig. 3:**
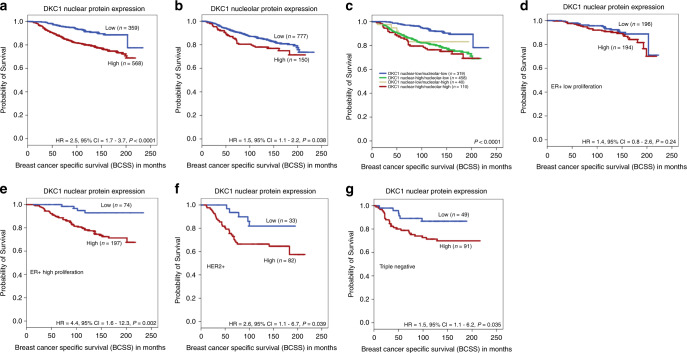
This figure shows the association between DKC1 protein expression and breast-cancer-specific survival (BCSS) as follows: **a** DKC1 nuclear expression and BCSS, **b** DKC1 nucleolar expression and BCSS **c** combinatorial DKC1 protein expression and BCSS **d** DKC1 and BCSS in oestrogen receptor (ER)+ low proliferation tumours **e** DKC1 and BCSS in (ER)+ high proliferation tumours **f** DKC1 and BCSS human epidermal growth factor receptor 2 positive (HER2+) tumours **g** DKC1 and BCSS of triple negative tumours in the studied cohort.

Both high DKC1 nuclear and nucleolar protein expressions, when assessed individually, were associated with poor outcome (HR = 2.5, 95%CI = 1.7–3.7; *p* < 0.0001 and HR = 1.5, 95%CI = 1.1–2.2; *p* = 0.038, respectively) Fig. 3a, b. When the analysis was limited to molecular subtypes, high expression of DKC1 nuclear protein was significantly associated with poor outcome in ER+ high proliferation tumours (HR = 4.4, 95% CI = 1.6–12.3; *p* = 0.002), HER2+ tumours (HR = 2.6, 95% CI = 1.1–6.7; *p* = 0.039) and TNBC (HR = 1.5, 95% CI = 1.1–6.2; *p* = 0.035) Fig. 3d–g. However, no significant association of DKC1 nucleolar protein expression was identified with outcome in BC subtypes (*p* > 0.05).

For further analysis, combinatorial DKC1 protein expression groups were created [i.e. low nuclear/low nucleolar, high nuclear/low nucleolar expression, low nuclear/high nucleolar and high nuclear/high nucleolar]. A significant difference in patient survival was observed between these four groups, where tumours with low nuclear and low nucleolar DKC1 expression showed the best outcome, whereas the tumours with high nuclear and high nucleolar expression showed the worst outcome (*p* < 0.0001) Fig. 3c.

The multivariate Cox-proportional models, including other prognostic covariates such as tumour size, grade and nodal stage, showed that DKC1 nuclear, combinatorial protein expression and *DKC1* mRNA in the METABRIC dataset were independent predictors for poor prognosis in whole cases (*p* = 0.001, HR 2.037, 95% CI = 1.373–3.023, *p* = 0.003, HR 2.746, 95% CI = 1.484–5.083 and *p* = 0.006, HR 1.316, 95% CI = 1.097–1.579) as shown in Table 3 and Supplementary Table 5.

**Table 3:** Multivariate Cox regression analysis results for predictors of breast-cancer-specific survival in Nottingham cohort.

Models	Parameters	Hazard ratio (HR)	95% confident interval (CI)		Significance *P* value	Adjusted *P* value
			Lower	Upper		
(A)	DKC1 nuclear protein expression	2.037	1.373	3.023	0.000411	**0.001**
	Tumour size	1.445	1.047	1.995	0.025	**0.025**
	Nodal stage	2.036	1.664	2.492	5.1 × 10^−12^	**<0.0001**
	Grade	1.426	1.079	1.885	0.013	**0.026**
(B)	DKC1 nucleolar protein expression	1.218	0.832	1.802	0.325	0.325
	Tumour size	1.481	1.072	2.045	0.017	**0.034**
	Nodal stage	2.068	1.69	2.532	1.9 × 10^−12^	**<0.0001**
	Grade	1.52	1.148	2.012	0.003	**0.009**
(C)	DKC1 combinatorial protein expression	2.746	1.484	5.083	0.001	**0.003**
	Tumour size	1.642	0.922	2.922	0.092	0.184
	Nodal stage	2.018	1.411	2.888	0.000123	**0.0005**
	Grade	1.037	0.637	1.69	0.883	0.883

*P* values in bold means statistically significant.

In addition, high DKC1 nuclear protein expression was significantly associated with shorter distant metastases-free survival (DMFS) (HR = 2.1, 95% CI = 1.5–2.9; *p* < 0.0001). Likewise, combinatorial protein expression was associated with shorter DMFS (HR = 1.3, 95% CI = 1.1–1.4; *p* = 0.001) (Supplementary Fig. 4).

## Discussion

In malignant tumours, the number and size of nucleoli are usually an indication of the rate of ribosome production, which is regarded as a major metabolic requisite for cell growth and proliferation.^39,40^ Nucleolar function and size are directly related to cell doubling time in cancer cells and quantitative morphometric evaluation of nucleolar size was considered as a cytological parameter of the tumour cells proliferation rate.^4,41^ In this study, we assessed NP in the TCGA breast cancer dataset as previously described,^4^ and used two greedy algorithms information theory and validated it using LASSO regression test^22–24^ to identify genes driving NP. This demonstrated that out of the 20,339 genes investigated, *DKC1* was the top differentially expressed gene. Then DKC1 expression was evaluated at the proteomic, transcriptomic and genomic levels in large cohorts of invasive BC.

There were significant associations between high *DKC1* mRNA in TCGA breast cancer dataset and *DKC1* protein expression in Nottingham cohort with high nucleolar scoring.^4^ These findings supported our hypothesis that DKC1 plays a role in the nucleoli appearance and size likely through its mechanism in ribosomal biogenesis.

Our results also showed positive correlations between nuclear and nucleolar DKC1 protein expression in the breast tumour cells. It was reported that newly synthesised DKC1 initially localises to the nucleoplasm, followed by consecutive translocation to the nucleoli and the nuclear Cajal bodies. Usually, colocalisation of DKC1 on the nuclear Cajal bodies occurred only when it had already accumulated in the nucleoli.^42^ DKC1 is involved in the pseudouridination and processing of small spliceosomal RNAs through its binding to H/ACA small Cajal body RNAs.^43^

The current study confirms the significant association between the high expression of DKC1, at both protein and mRNA levels, and clinicopathological parameters characteristics of poor prognosis and with shorter survival; findings which are in-line with other studies.^15,44,45^ Some studies have also confirmed that *DKC1* overexpression is involved in tumorigenic processes and has prognostic value in numerous types of cancer.^19,21,46^ The association between *DKC1* mRNA and shorter survival was identified in both METABRIC and TCGA cohorts. Moreover, our analysis revealed that the prognostic significance of DKC1 protein and mRNA in BC was independent of other variables, demonstrating its potential clinical relevance in improving survival rate prediction.

When BC molecular subtypes were considered, the significant association between DKC1 protein and poor patient outcome was observed in the ER+ high proliferation (i.e. luminal B), HER2+ and TNBC classes whereas the high mRNA expression was only limited to the luminal B subtype. The most common type of BC constituting nearly 55–70% is the ER+/luminal tumour, and those tumours are variable in terms of recurrence, mortality rates and disease prognosis.^47,48^ These observations further endorse DKC1 functions in playing crucial roles in tumour growth and progression

DKC1 performs two fundamental functions for cell proliferation. First, DKC1 is a component of the H/ACA small nucleolar ribonucleoprotein particles (snoRNPs) involved the pseudouridylation of ribosomal RNA (rRNA) molecules and necessary for their processing. Second, it is required for telomerase activity by stabilising the telomerase RNA component.^11^ The faster the rate of cell proliferation, the higher the demand for protein production, which is compatible with increased rRNA synthesis.^49,50^ It has been reported that any dysregulation of DKC1 levels results in defects of ribosome biogenesis and a reduction of rRNA pseudouridylation, which in turn hinders the normal ribosome rRNA processing rate.^51^ For instance, Montanaro et al. have demonstrated that reduced *DKC1* gene expression by specific RNA interference in BC cell lines resulted in a reduction of rRNA pseudouridylation, which subsequently effected the survival of proliferating cells.^52^ The role of DKC1 in mitosis was also confirmed,^11^ where dyskerin was identified as one of seventy genes which correlated with the development of aneuploidy. Alawi et al. have demonstrated that dyskerin expression peaks during G_2_/M and loss of dyskerin function has a widely disruptive effect on mitosis and triggers the spindle-assembly checkpoint.^13^ Our findings showed that high DKC1 expression was significantly associated with proliferation as assessed by Ki67 labelling index, which was also observed in other studies in BC^52^ hepatocellular carcinoma^15^ and prostate cancer,^19^ confirming that DKC1 is critical for mitotic progression and proliferation in these cancers.

*DKC1* is the direct and conserved transcriptional target of c-Myc,^53^ which explains the strong correlation between its upregulation and active cell proliferation.^54^ In our study, we observed a significant positive association between *DKC1* and *c-Myc* in mRNA expression. Previous studies have demonstrated that tumour oncogene *c-Myc* controls the transcription of *DKC1* gene in addition to other proteins, which are required for rRNA processing.^9,55^ *TP53* mutations were also highly prevalent in breast tumours with high *DKC1* mRNA expression in METABRIC. There is mounting evidence that the usual increase of ribosome biogenesis (one of the main functions of *DKC1*) in cancer cells is the consequence of frequent alterations of two major tumour suppressors*, TP53* and retinoblastoma (RB) genes.^55^ In addition, tumours with altered *p53* and/or retinoblastoma protein *pRb* functions are characterised by significantly larger/more conspicuous nucleoli than tumours with normally functioning *p53* and *pRb*.^56^

We further investigated the association of *DKC1* expression with other H/ACA ribonucleoproteins, *NHP2*, *NOP10* and *GAR1*, which play important roles in disease progression. *DKC1*, *NHP2* and *NOP10* form a core trimer that directly binds to H/ACA RNAs. The three proteins are interdependent with each other for stability and also regulate constancy of the bound RNAs.^57^ *GAR1* binds only to *DKC1* and is needed for a proper functioning of the H/ACA RNPs, but its absence does not reduce the stability of the rRNA.^58^ These findings confirmed the significant positive correlation between H/ACA ribonucleoproteins and *DKC1* in BC.^43^ Alterations in *DKC1* expression will potentially disrupt the biogenesis of H/ACA pathway and consequently affect ribosome synthesis and impair cell proliferation.

In the last decade, there have been a few attempts to construct *DKC1* inhibitors. However, one in silico study successfully determined a small molecule inhibitor (Pyrazofurin) that exerted an ability to weaken the pharmacological and physiological activities of DKC1 through inhibiting its function in pseudouridylation of rRNA. Although Pyrazofurin failed to progress Phase 2 clinical trials; however, its chemical structure should continue to be exploited as a pharmacokinetic model to develop a potent, effective and safe *DKC1* inhibitor that may eventually be used for BC highly expressing *DKC1*.^59^

A few limitations of this study findings are worth mentioning. On one hand, the semi-quantitative H-score method used to evaluate the immunohistochemical protein expression in the Nottingham cohort, might be regarded as having substantial subjectivity. This was addressed by double scoring a subset of the cancers to ensure the reproducibility and liability of the procedure. On the other hand, only one representative TMA core from each tumour tissue was arrayed and scored instead of considering replicates to express the tumour heterogeneity. This was due to the limited tissue resources in our biobank. However, to overcome the issue, 20 full face sections of randomly selected breast cancer cases were stained, prior to TMA application, with DKC1 antibody to assess the staining homogeneity and to evaluate the pertinence of using tissue microarrays (TMAs). These showed homogeneously distributed DKC1 expression, deeming the use of TMA to assess DKC1 an appropriate tissue platform, to mitigate the limited resources as well as testing the hypothesis a large BC cohort. Finally, the ‘weak’ but significant correlation between nuclear and nucleolar expression of DKC1 might also be regarded as a weakness point in the study. This might be due to the subjectivity of the method, which has been used in determining NP in our previous study.^4^

## Conclusion

This study reveals a significant correlation between the morphological features of NP and an underlying molecular and protein description (DKC1). The importance of DKC1 was demonstrated in three independent datasets where each dataset contributed to the description of DKC1 from different perspectives. DKC1 is significantly associated with high nucleolar score and with poor prognostic characteristics and poor patients’ outcome. Overexpression of DKC1 appears to play a role in the proliferation and progression of the aggressive BC subtypes including the luminal B, TNBC and HER2 molecular subtypes. Findings here encourage further investigation of DKC1 as it might relate to guiding targeted therapies and to evaluate its role in response to chemotherapy.

## Supplementary information

Supplementary material

## Data Availability

The authors confirm the data that have been used in this work are available on reasonable request.
