# Supplementary material for: The nucleolar-related protein Dyskerin pseudouridine synthase 1 (DKC1) predicts poor prognosis in breast cancer
Source: Br J Cancer. 2020 Sep 1;123(10):1543–52. doi: 10.1038/s41416-020-01045-7 (PMC7653035; doi:10.1038/s41416-020-01045-7)
Supplement: Supplementary file 1 — Supplementary material [file 41416_2020_1045_MOESM1_ESM.docx]

**Supplementary Tables**

**Supplementary Table 1:** Clinicopathological characteristics of the cases in both METABRIC and Nottingham series.

| **Parameter** | **METABRIC series**  **Number of cases (%)** | **Nottingham series**  **Number of cases (%)** |
| --- | --- | --- |
| **Patient Age**  ≤ 50 years  > 50 years | 383 (20)  1556 (80) | 348 (37)  580 (63) |
| **Tumour size**  ≤ 2cm  > 2cm | 622 (32)  1331 (68) | 353 (38)  575 (62) |
| **Tumour grade**  1  2  3 | 170 (9)  770 (41)  952 (50) | 105 (11)  392 (42)  432 (47) |
| **Stage**  1  2  3 | 1035 (53)  622 (31)  316 (16) | 572 (62)  261 (28)  95 (10) |
| **Vascular invasion**  Definite  Negative/Probable | Not available | 293 (32)  635 (86) |
| **Oestrogen receptor status**  Positive  Negative | 1506 (76)  474 (24) | 748 (80)  182 (20) |
| **Progesterone receptor status**  Positive  Negative | 1040 (53)  940 (47) | 565 (61)  362 (39) |
| **HER2 receptor status**  Positive  Negative | 247 (13)  1733 (87) | 115 (12)  815 (88) |
| **Nottingham prognostic index (NPI)**  Good  Moderate  Poor | 680 (34)  1101 (56)  199 (10) | 281 (31)  489 (53)  150 (16) |
| **Survival status**  Alive  Dead | 1071 (68)  505 (32) | 718 (77)  211 (23) |

**Supplementary Table 2:** Clinicopathological associations of *DKC1* mRNA expression in TCGA dataset of breast cancer.

| **Parameters** | ***N* (%)** | **Mean Rank** | ***p*-value** | **Adjusted *p*-value** |
| --- | --- | --- | --- | --- |
| **Patient Age**  ≤50  >50 | 231(27)  623 (73) | 466.6  413.02 | 0.005 | **0.01** |
| **Tumour size**  ≤2cm  >2cm | 239 (28)  615 (72) | 386.8  443.3 | 0.003 | **0.009** |
| **Lympho-vascular Invasion**  Negative  Positive | 559 (65)  295 (35) | 407.7  464.9 | 0.001 | **0.004** |
| **Lymph node stage**  Negative  Positive | 426 (50)  423 (50) | 432.1  417.8 | 0.4 | 0.4 |
| **Tumour Grade**  Grade 1  Grade 2  Grade 3 | 89 (11)  375 (46)  352 (43) | 237.9  310.7  555.8 | 1.1 X10^-54^ | **<0.0001** |
| **Nucleoli**  Score 1  Score 2  Score 3 | 289 (49)  232 (40)  66 (11) | 214.1  348.3  453 | 1.9 X10^-32^ | **<0.0001** |

*P* values in bold means statistically significant

**Supplementary Table 3:** Correlation of *DKC1* mRNA expression with the expression of other genes in METABRIC & TCGA breast cancer datasets.

|  | **METABRIC** | | | **TCGA** | | |
| --- | --- | --- | --- | --- | --- | --- |
| **Genes** | **Correlation Coefficient** | ***p*-value** | **Adjusted *p-*value** | **Correlation Coefficient** | ***p*-value** | **Adjusted *p*-value** |
| *c-Myc* | 0.299 | 2.9 X10^-42^ | **<0.0001** | 0.341 | 1.2 X10^-24^ | **<0.0001** |
| *MKI67* | 0.509 | 3.3 X10^-131^ | **<0.0001** | 0.419 | 1.5 X10^-37^ | **<0.0001** |
| *GAR1* | 0.401 | 1.5 X10^-77^ | **<0.0001** | 0.348 | 1.2 X10^-25^ | **<0.0001** |
| *NOP10* | 0.071 | 0.001 | **0.001** | 0.151 | 0.000009 | **<0.0001** |
| *NHP2* | 0.165 | 1.3 X10^-13^ | **<0.0001** | 0.171 | 5.2 X10^-7^ | **<0.0001** |

*P* values in bold means statistically significant

**Supplementary Table 4:** Association of *DKC1* mRNA expression and other molecular biomarkers in METABRIC and in TCGA breast cancer series.

|  | ***DKC1* mRNA (METABRIC)** | | | | ***DKC1* mRNA (TCGA)** | | | |
| --- | --- | --- | --- | --- | --- | --- | --- | --- |
| **Parameters** | **Number (%)** | **Mean Rank** | ***p-value*** | **Adjusted *p*-value** | ***N*umber (%)** | **Mean Rank** | ***p-value*** | **Adjusted *p*-value** |
| **Oestrogen Receptor**  Negative  Positive | 474 (24)  1506 (76) | 1374.8  869.5 | 3.3 X10^-63^ | **<0.0001** | 185 (22)  639 (78) | 620.9  352.1 | 1.1 X10^-41^ | **<0.0001** |
| **Progesterone Receptor**  Negative  Positive | 940 (47)  1040 (53) | 1144.2  851.6 | 5.9 X10^-30^ | <**0.0001** | 272 (33)  546 (67) | 547.7  340.6 | 1.3 X10^-31^ | **<0.0001** |
| **HER2 status**  Negative  Positive | 1733 (87)  247 (13) | 966.7  1157.7 | 8.9 X10^-7^ | **<0.0001** | 567 (81)  133 (19) | 348.3  359.9 | 0.548 | 0.548 |
| **Triple negative status**  Non-triple negative  Triple negative | 1660 (84)  320 (16) | 908.1  1418.2 | 2.6 X10^-48^ | **<0.0001** | ***N/A*** | ***N/A*** | ***N/A*** | ***N/A*** |
| **TP53 mutations**  Wild type  Mutation | 721 (88)  99 (12) | 391.3  550.7 | 3.4 X10^-10^ | **<0.0001** | ***N/A*** | ***N/A*** | ***N/A*** | ***N/A*** |

*P* values in bold means statistically significant

**Supplementary Table 5**: Multivariate Cox regression analysis results for predictors of Breast Cancer Specific Survival in METABRIC breast cancer dataset.

| **Parameters** | **Hazard ratio**  **(HR)** | **95% confident interval (CI)** | | **Significance**  ***p*-value** | **Adjusted *p-*value** |
| --- | --- | --- | --- | --- | --- |
|  |  | **Lower** | **Upper** |  |  |
| ***DKC1*mRNA** | 1.316 | 1.097 | 1.579 | 0.003 | **0.006** |
| **Tumour Size** | 1.604 | 1.282 | 2.008 | 0.000036 | **0.0001** |
| **Stage** | 1.899 | 1.685 | 2.141 | 8.6 X10^-26^ | **<0.0001** |
| **Grade** | 1.282 | 1.084 | 1.516 | 0.004 | **0.004** |

*P* values in bold means statistically significant

**Supplementary Figures**


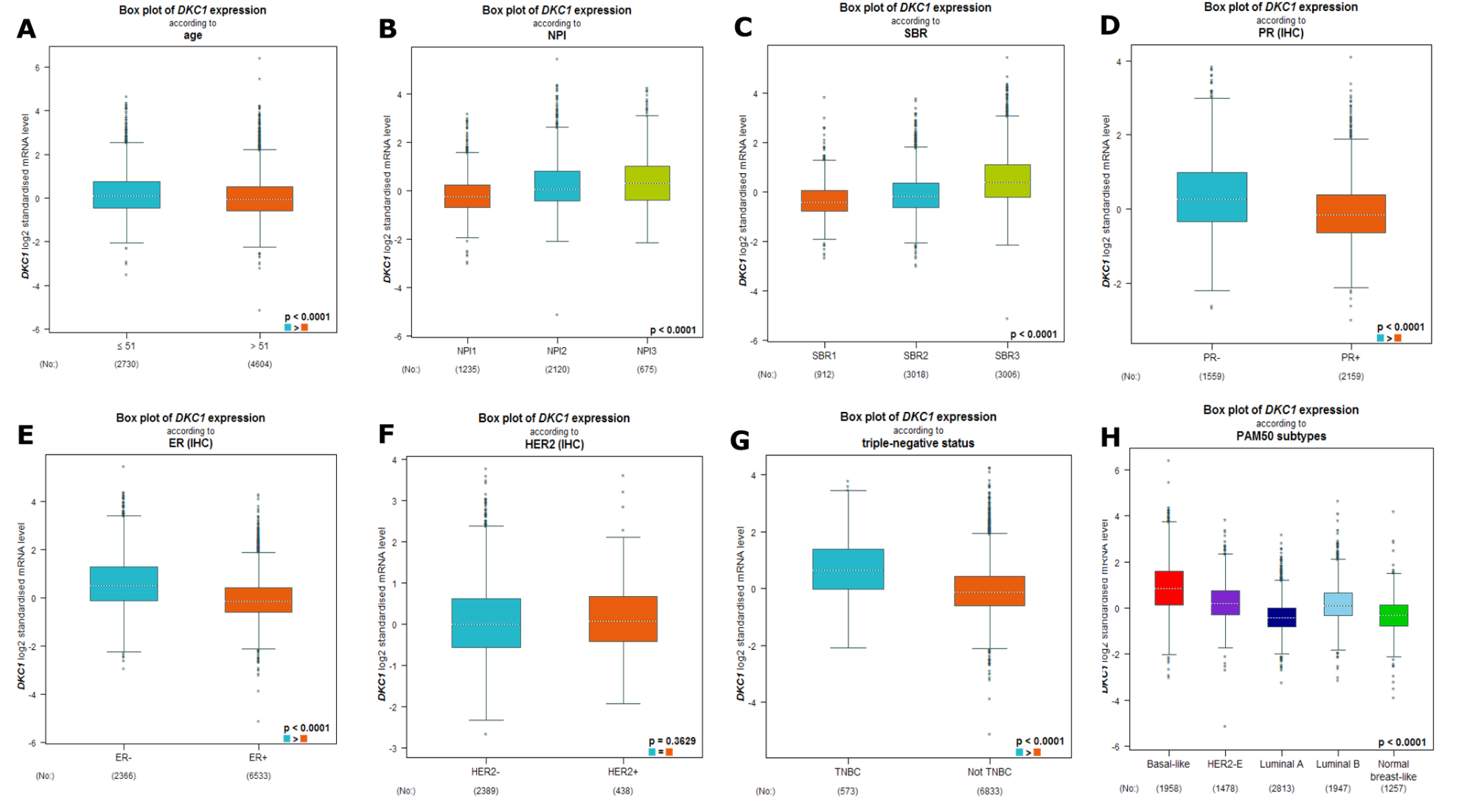


**Supplementary figure 1:** *DKC1* gene expression and its association with: **A.** patient’s age **B.** Nottingham prognostic index, **C.** tumour grade, **D.** PR status, **E.** ER status, **F.** HER2 status, **G**. Triple Negative status, **H.** PAM50 subtypes using Breast Cancer Gene-Expression Miner.

**
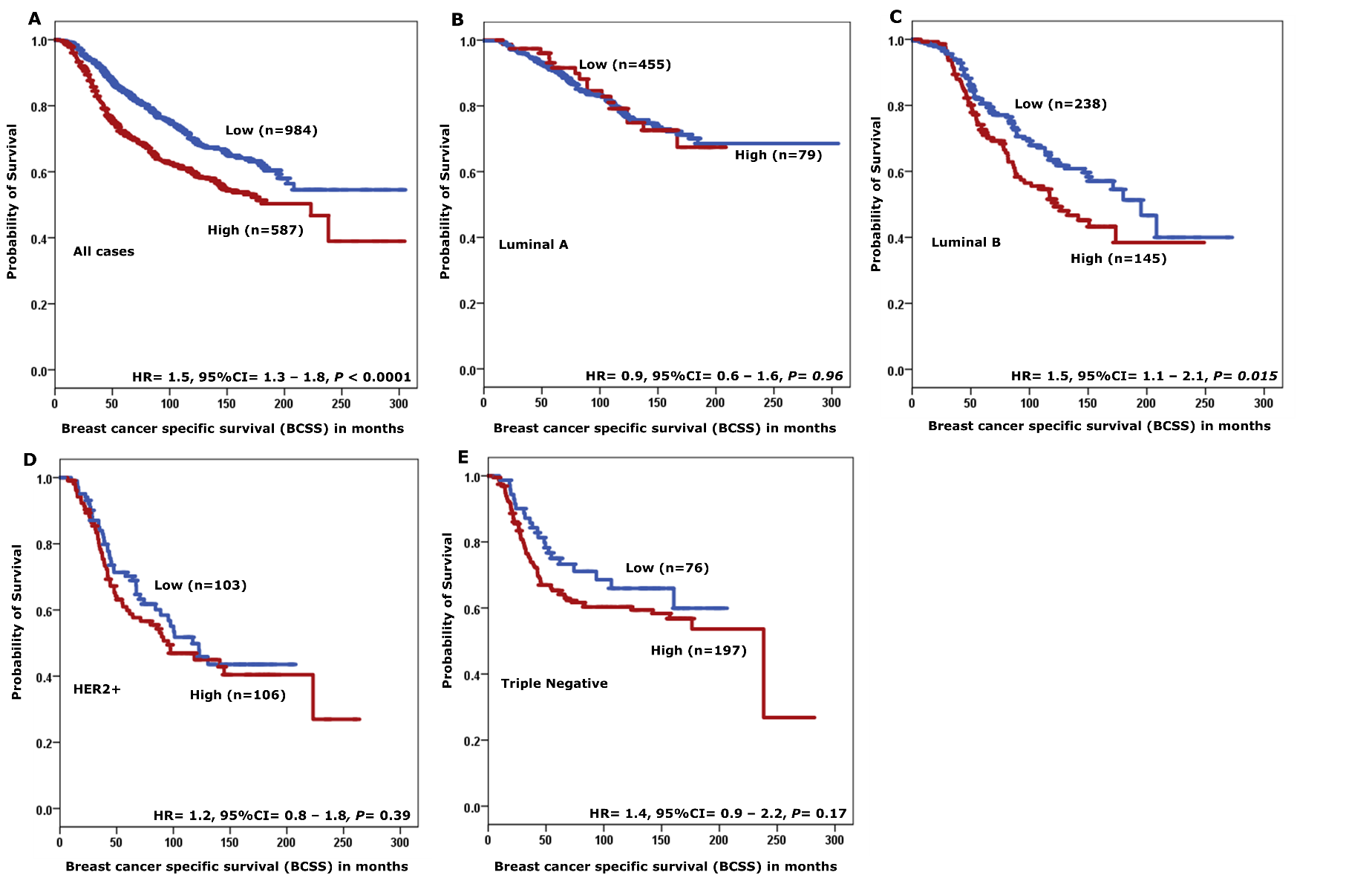
**

**Supplementary figure 2:** *DKC1* mRNA expression and BCSS in the METABRIC cohort.**A** *DKC1* and breast-cancer-specific survival (BCSS) in all cases. **B** *DKC1* and BCSS in luminal A tumours. **C***DKC1* and BCSS in luminal B tumours. **D***DKC1* and BCSS in in human epidermal growth factor receptor 2 (HER2+) tumours. **E** *DKC1* and BCSS in triple negative tumours.


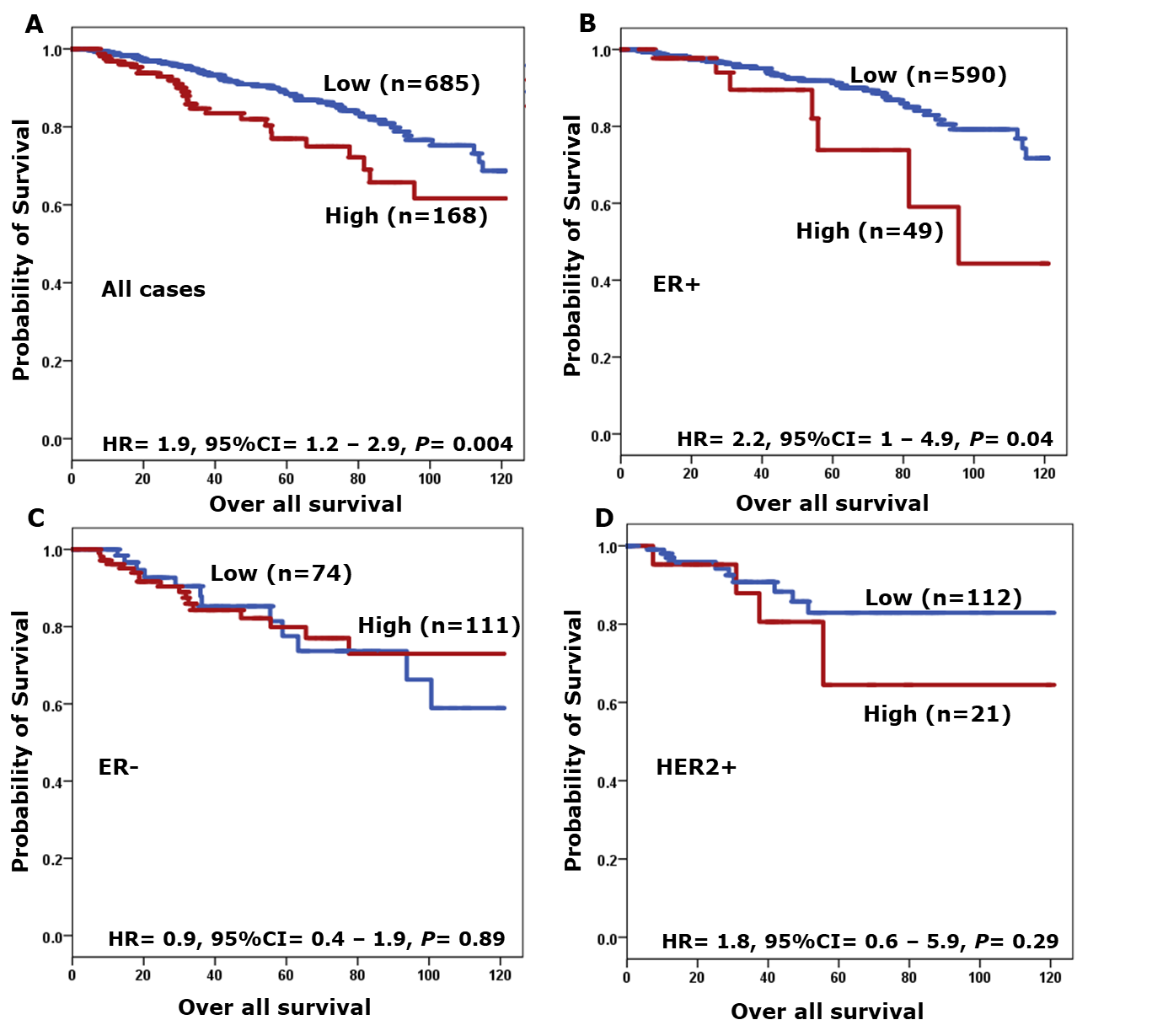


**Supplementary figure 3:** *DKC1* mRNA and breast cancer patient outcome in **A.** all cases **B.** ER+ tumours **C.** ER- tumours **D.** HER2+tumours in TCGA breast cancer dataset.

**
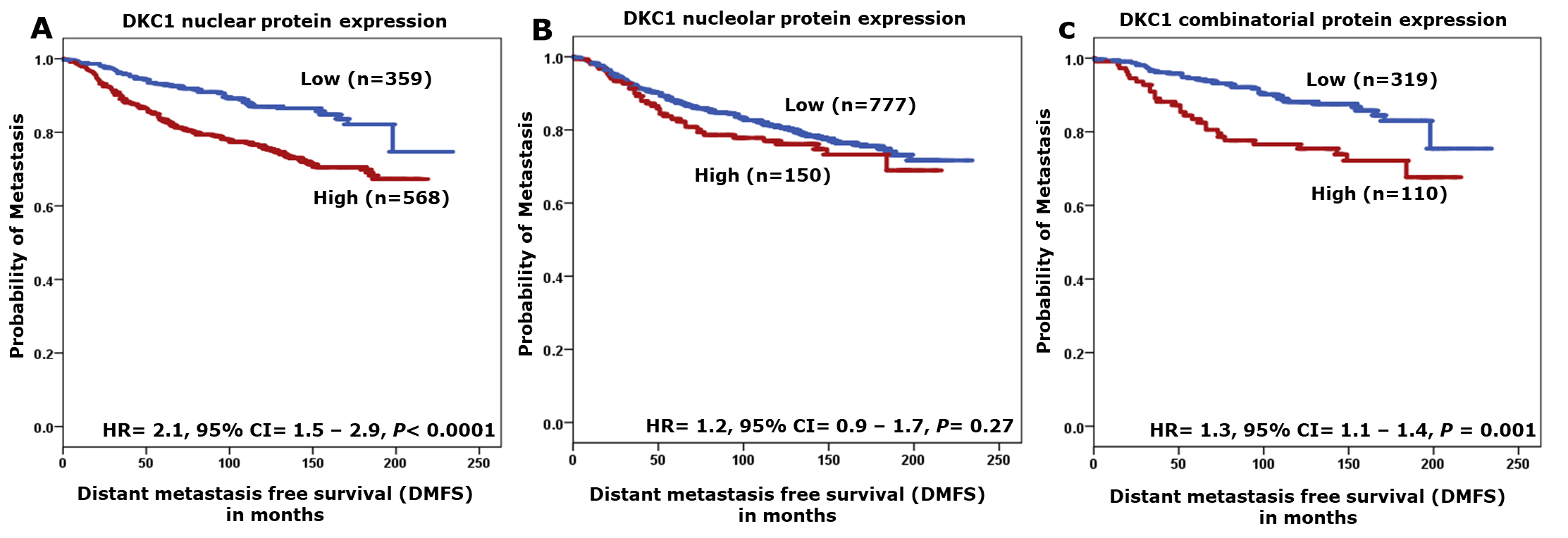
**

**Supplementary figure 4:** DKC1 protein expression and distant metastasis free survival (DMFS). **A** DKC1 nuclear expression and DMFS. **B** DKC1 nucleolar expression and DMFS. **C** combinatorial DKC1 protein expression and DMFS in in the studied cohort
